# Adult sedation and analgesia in a resource limited intensive care unit – A Systematic Review and evidence based guideline

**DOI:** 10.1016/j.amsu.2021.102356

**Published:** 2021-04-30

**Authors:** Netsanet Temesgen, Bsazinew Chekol, Tadesse Tamirie, Denberu Eshetie, Nigussie Simeneh, Abatneh Feleke

**Affiliations:** a Debre Tabor University, College of Health Sciences, School of Medicine, Department of Anesthesia, Ethiopia; b University of Gondar, College of Medicine and Health Sciences, Department of Anesthesia and Critical Care, Ethiopia

**Keywords:** Sedation and analgesia in the ICU, ICU Patients, ICU Analgesia, ICU Sedation

## Abstract

**Background:**

Sedation and analgesia are essential in the intensive care unit in order to promote control of pain, anxiety, prevent loss of materials, accidental extubation and improve the synchrony of patients with ventilator. However, excess of these medications leads to an increased morbidity and mortality, and thus demands protocol.

**Methods:**

Preferred Reporting Items for Systematic Reviews and the Meta-Analysis Protocol have been used to undertake this review. Pub Med, Cochrane Library, and Google Scholar search engines were used to find up-to-date evidence that helps to draw recommendations and conclusions.

**Results:**

In this Guideline and Systematic Review, we have used 16 Systemic Review and Meta-Analysis, 3 Evidence-Based Guidelines and 10 RCT Meta-Analysis, 6 Systemic Reviews of Non-randomized Studies, 8 Randomized Clinical Trials, 11 Cohort Studies, 5 Cross-Sectional Studies and 1 Case Report with their respective study descriptions.

**Discussion:**

Analgesia, which as a sedation basement can reduce sedative use, is key aspect of treatment in ICU patients, and we can also conclude that an analgesic sedation regimen can reduce the occurrence of delirium by reducing sedatives. The aim of this guideline and the systematic review is to write up and formulate analgesia-based sedation for limited resource settings.

**Conclusions:**

Analgesia and sedation are effective in critically ill patients; however, too much sedation is associated with longer periods of mechanical ventilation and longer duration of ICU stay. Poorly managed ICU patients have a delirium rate of up to 80%, increased mortality, longer hospital stays, higher hospital costs and bad long-term outcomes.

## Highlights

• Critically ill patients shall be awake, alert without pain, anxiety and delirium.
• Analgesia and sedation in the ICU shall be given as per needed after determined they are in need of.
• Sedation breaks are paramount important as equal as spontaneous breathing trials.
• Unnecessary deep sedations will increase hospital and personnel costs by increasing length of ICU stay.

## 1 Background

Sedation and analgesia are essential components in the management of all critically ill patients [1]. Those requiring mechanical ventilation and the main indications for use include to alleviate patient discomfort, anxiety and agitation, cause amnesia, promote mechanical ventilation, prevent the displacement of endotracheal tubes, and decrease cell metabolism [2].As a result, deep sedation was commonly used until a patient was able to breathe without assistance [3], and developments over the past 30 years, including microprocessor-controlled ventilators that synchronize with patients' own respiratory efforts and new, shorter-acting sedative and analgesic medications, have drastically changed the way ICU patients are treated with various treatments (most notably endotracheal intubation and invasive mechanical ventilation) that are experienced or interpreted [4].

Pain is a common experience for most ICU patients and failing to recognize that pain also contributes to agitation. It is the most common memory that patients with their ICU stay and inadequate analgesia and anxiety can precipitate accidental removal of endotracheal tubes or intravascular catheters used to track or administer life-sustaining medications. As a result, sedatives and analgesics are now becoming one of the most widely used and used medications in ICU, as equally important ideas have been mentioned that early detection of pain, sedation, sedation, and delirium are problems that if undetected and untreated, are distressing to patients and associated with increased ICU morbidity and mortality [3,5].

Analgesia and sedation are important components in the treatment of patients in the intensive care unit (ICU) in order to facilitate management of pain, anxiety and agitation, avoid failure of equipment, involuntary extubation and enhance the coordination of patients with mechanical ventilation. However, excess of these drugs contributes to an increase in morbidity and mortality [6]. Ideal treatment would rely on the implementation of clinical and pharmacological interventions, driven by scales and guidelines, but the identification and control of pain by various scholars is difficult in Intensive Care Units (ICUs) due to a variety of conflicting factors correlated with short-and long-term effects of inadequate pain relief leading to increased adverse outcomes [2,7].

Sedatives are very common in ICU settings and cannot be thought of as sedative-free ICU sedations, and these sedatives can alleviate patient distress and stress levels, improve care delivery and ensure protection, and these sedatives are prescribed to 85% of Intensive Care Unit (ICU) patients, including intravenous benzodiazepines and propofol are the most commonly used sedatives [8,9]. However these agents are associated with over-sedation in 40–60% of patients, which can lead to prolonged intubation, delirium and drug-induced hypotension [4].

Evidences show that newer volatile anesthetic agents are associated with faster extubation times, improved cardiovascular safety with no end-organ toxicity relative to our normal intravenous agents for short-term critical care sedation. The use of this volatile agent in the ICU is a novel technique that uses a specialized distribution and scavenging procedure that involves personnel training and cultural acceptance and sedation protocols and daily sedation interruption does not appear to vary in comparison to the majority of the findings analyzed [5].

Proper sedation is an important component in the care of critically ill patients requiring mechanical ventilation [10]. Deep sedation levels are associated with many negative effects, such as increased mechanical ventilation time longer ICU stay, delirium, memory disruptions, and higher short-and long-term mortality. In ICU patients, especially those with mechanical ventilation, the rate of delirium is as high as 80%, in addition to increased mortality, longer hospital stays, higher hospital costs and poor long-term outcomes are normal [6,11]. These and other deleterious effects of deep sedation can be minimized by employing a strategy of sedation protocols that target lighter sedation levels and the daily interruption of sedative infusion [12]. The results of these techniques were evaluated in two systematic reviews in which the included research control groups consisted of patients who received “usual treatment for sedation of patients with mechanical ventilation [6].

Analgesia, which as a sedation basement may reduce the amount of sedatives used is a key and key component of treatment in the management of ICU patients, and we may therefore conclude that an analgesic sedation protocol can reduce the incidence of delirium due to a reduction in the amount of sedatives used [4,13].

Specific physiological changes that critically ill patients experience can have a direct impact on the pharmacology of medications, possibly contributing to discrepancies in response between patients. Objective measures of pain, sedation and anxiety have been validated for use in the ICU for the evaluation and titration of drugs [14,15]. An evidence-based approach for the administration of these medications will lead to changes in patients' short-and long-term outcomes. In this guideline, we have reviewed a variety of literature and innovations in the field of ICU sedation to include an up-to-date perspective on procedures for the treatment of mechanically ventilated adult ICU patients [16,17].

## 2 Methods

This Evidence Based Guideline and Systematic Review is presented by Preferred Reporting Items Reviews and Meta-Analysis (PRISMA). The level of proof and advice was assessed on the basis of WHO Evidence of Good Clinical Practice (GCP) and evaluated on the basis of different assessment checklists to categorize them to level 1 (Meta-analysis and systematic review of RCTs, Randomized control trials, Evidence based guide lines), level 2 (systematic review of Well-designed cohort studies) and level 3(Non analytical studies).

The terms ICU sedation and analgesia, ‘ICU patients,’ ‘ICU analgesia’ and ‘ICU sedation’ have been used in different combinations. After a fair amount of data has been obtained, the assessment and evaluation of the consistency of the research using a different institutional assessment checklist was used to categorize the evidence.

**Fig. 1:**
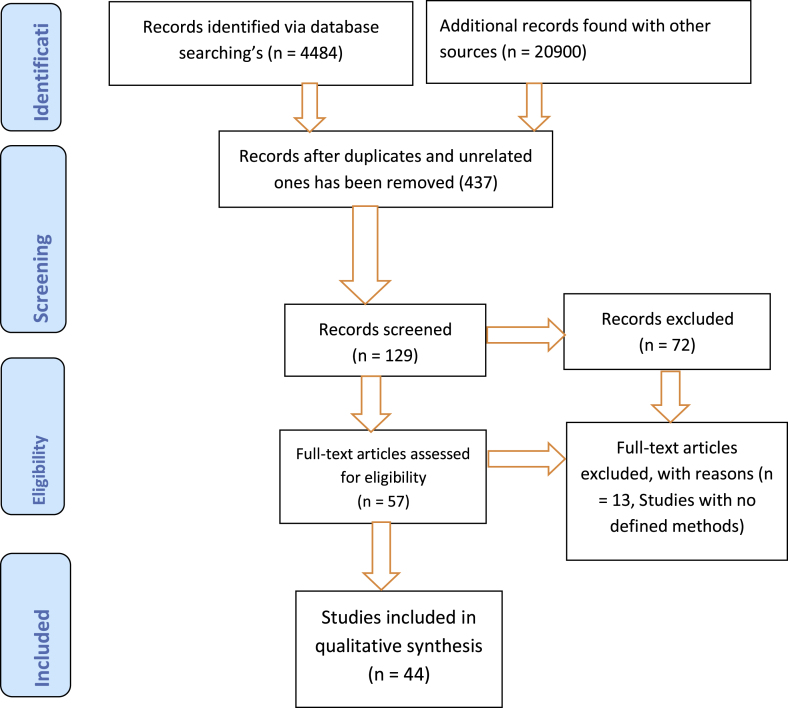
PRISMA flow diagram for the searched and used articles.

A total of 44 literatures (16 Met analysis and Systematic reviews, 8 RCTs, 11 Cohort, 3 Guidelines, 5urvey**s** and 1 case report) were considered and used in this Guideline after they have been filtered and analyzed accordingly (Fig. 1). In this review we have included only full text articles which have been written in English language and we excluded studies with no defined methods and published before the year 2000 GC.

Finally conclusions and recommendations are made by balancing the benefits and drawbacks of alternative treatment options for sedation and analgesia protocols in the ICU. Best possible Conclusions were eventually drawn from the literature on the basis of their strength of evidence and recommendations for sedation and analgesia in critically ill adult ICU patients (Table 1).

**Table 1:** Level of evidence and degrees of recommendations.

Level	Type of Evidence	No of Articles	Degrees of Recommendations
1a	Meta analyses, systematic reviews of randomized controlled trails	10	Strongly recommended and directly applicable
1 b	Systematic review of non-randomized controlled trails	6	Highly recommended and directly applicable
1c	Randomized Controlled Trails (RCTs)	8	Recommended and applicable
2a	Evidence based Guidelines	3	Extrapolated evidence from other studies
3a	Non analytic studies such as Cohort, Surveys, case reports and case series	17	Extrapolated Evidence from other studies

Good clinical practice, GCP, WHO, 2011.

This systematic review and met analysis is registered at www.research registry with ID of 6620 and available at https://www.researchregistry.com/browse-the registry#home/registration details/603cbc8873c40d001b3a44f1/.

## 3 Results

In this Evidence Based Guideline and Systematic Review, we reviewed 16 SR and MA, 8 RCTs 3 evidence-based recommendations, 11 cohort studies, 5 cross-sectional studies and 1 case report with their respective research details and core findings (Table 2).

**Table 2:** Summary of articles used for the development of this Systematic review and evidence based guideline.

SN	Authors and Publication year, Follow up duration	Title of the articles	Study Participants	Results/Recommendations
1	Ahlers et al., 2008,90 Days	Comparison of different pain scoring systems in critically ill patients in a general ICU- A prospective Cohort	113 Patients	In ventilated patients, BPS can only be used in combination with the NRS nurse to assess pain levels in the absence of unpleasant stimuli.
2	Baron et al., 2015,365 Days	Managements of Delirium &Agitation-An Evidence Based consensus and Guideline	284 Studies	Sedation shall be performed with a combination of hypnotic and analgesics
3	Barr J et al., 2013,210 Days	Prevention and Control of delirium- An evidence based guideline	472 Studies	Early detection and treatment of potential underlying causes of agitation and anxiety is important for ICU sedation.
4	Brush et al., 2009,395 Days	Sedation and analgesia for the mechanically ventilated patient- A Randomized Controlled Trials	92 Patients	Mechanically ventilated patients in the intensive care unit routinely need sedative and analgesic medicine to relieve pain and anxiety.
5	Burry L et al., 2014,90 Days	Daily sedation interruptions vs. no sedation protocols in the ICU-A systematic review and met analysis	9 RCTs and 1282 Patients	Light sedation is recommended so that patients are responsive and able to communicate and daily interruption of sedation is stimulated.
6	Dale et al., 2014,730 Days	Improved analgesia, sedation, and delirium protocol associated with decreased duration of delirium and mechanical ventilation-A prospective Cohort	1483 Patients	Protocols for the administration of analgesia, sedation and delirium to critically ill, mechanically ventilated patients have been shown to improve outcomes but are not uniformly used.
7	Deffland et al., 2020,395 Days	Effects of pain, sedation and delirium monitoring on clinical and economic outcome-A retrospective cohort study	1323 Patients	Significant improvements in clinical outcome can be achieved by implementing effective strategies to optimize pain management, reduce sedative exposure, and prevent and treat delirium in ICU patients
8	Devabhakthunis et al., 2012,363 Days	Analgosedation: A paradigm shift in intensive care unit sedation practice-A systematic review and metanalysis	10 RCTs and 1155 patients	Analgosedation is an efficient and well-tolerated approach to ICU sedation treatment with better patient outcomes relative to sedative-hypnotic approaches.
9	Devlin et al., 2009, Follow up duration is not stated in the study,	Pharmacology of commonly used analgesics and sedatives in the ICU,benzodiazepines, propofol, and opioids-A Randomized controlled trail	206	Patients who are critically ill and have mechanical ventilation also need sedation and analgesic treatment to improve patient comfort, promote patient-ventilator coordination and optimize oxygenation.
10	Ely E Wesley,2003,48 Days	Monitoring sedation status over time in ICU patients-Reliability and validity of the Richmond Agitation-Sedation Scale-A prospective cohort study	313 Patients	RASS has been shown to be highly accurate and has extended the collection of pivotal sedation scores that are calculated by patients responding to verbal and physical stimulation by assisting with drugs.
11	Fraser Gl et al.,2007, Follow up duration is not stated in the study	Sedation and analgesia in the critically ill adult- A prospective Cohort	408	The approach to include analgesia-first and complemented by sedation-as-needs tends to improve patient outcomes in the ICU.
12	Fraser GL et al., 2013, Follow up duration is not stated in the study	Benzodiazepines Vs non benzodiazepines therapy- A systematic review and met analysis	6 RCTs and 1235 patients	Midazolam for short-term sedation only, lorazepam for long-term sedation, and Propofol for patients needing occasional awakening.
13	Hutton B et al., 2016/18,730 Days	Sedation strategies in the ICU- A systematic review and met analysis	54 RCTs	Protocolized sedation or daily sedation interruption is recommended.
14	Jareth et al., 2015, Follow up duration is not stated in the study	Use of volatile anaesthetics- A randomized Controlled Trials	60 Adult ICU Patients	Volatile anaesthetics have many pharmacological properties, making it suitable for extended use in ICU sedation.
15	Keoph SJ et al., 2015,365 Days	Analgesia based sedation in the ICU-Evidence based Guideline	145 Patients	Midazolam and fentanyl were the most commonly used sedation and analgesia medications during mechanical ventilation.
16	Kim HY et al., 2017 Follow up duration is not stated in the study	Volatile sedation in the ICU- A systematic review and met analysis	13RCTs and 1027 patients	Inhalational sedation enhances early recovery, decreased ICU stay and shortens mechanical ventilation.
17	Kress JP et al., 2002, Follow up duration is not stated in the study	Sedation and analgesia in the intensive care unit- A Randomized controlled Trail	80 Patients	Sedation is an important component of the care of patients who are mechanically ventilated and critically ill. There is currently a broad range of pharmacological agents available for the complex needs of this heterogeneous group of patients undergoing extended sedative administration.
18	Lavrentieva et al., 2017, Follow up duration is not stated in the study	Agitation, Sedation & Analgesia in the ICU- A systematic review and met analysis	64 RCTs	There is a substantial gap between the guidelines and clinical practice for the assessment of pain, sedation and delirium and mgt in the ICU setting.
19	Maclaren R et al., 2000,150 Days	Evaluation of empiric versus protocol‐based sedation and analgesia- A prospective cohort	72 empiric and 86 protocol therapy (158)	Compliance with the protocol decreased medication prices and increased sedation and analgesia safety for patients needing long-term sedation. Protocol-based therapy could have postponed extubation but did not postpone discharge of the ICU.
20	Martin et al., 2005,180 Days	Practice of sedation and analgesia in German intensive care units- A national survey	220 Participants	Propofol was the key short-acting agent used for sedation in ICUs and benzodiazepine midazolam was used for long-term sedation. Fentanyl and sufentanil have been used for analgesia.
21	Martin et al., 2006, Follow up duration is not stated in the study	Sedation and analgesia in German intensive care units: how is it done in reality? Results of a patient-based survey of analgesia and sedation- A postal survey	305 Participants	The fact that patients were more deeply sedated than expected by the therapist in all phases of sedation may be due to the low use of sedation scales and clinical procedure protocols or lack of experience in the use of these techniques.
22	Meiser et al., 2005 Follow up duration is not stated in the study	Inhalational anaesthetics in the ICU-Case Report	Two case reports	Most inhalation agents are poor analgesics and analgesia will be required, particularly in postoperative or trauma patients. Opioids, non-opioid medications and regional analgesia strategies can be mixed as needed.
23	Mukhopadhya et al., 2017,850 Days	Age related inverse doses in the ICU- An observational Cohort study	576 Patients	Possible interaction between propofol and fentanyl is an essential concern for elderly patients and Fentanyl can reduce the volume of the central compartment and thus the clearance of propofol.
24	Owen GD et al., 2019,2195 Days	International Analgesia, Sedation, and Delirium Practices - A prospective cohort study	14281 Patients	Analgesia and sedation practices have varied widely across international regions and have evolved dramatically over time. Opportunities for better treatment include increasing control of delirium, conducting SATs and decreasing use of sedation, in particular benzodiazepines.
25	Park GC et al., 2001, Follow up duration is not stated in the study	Balancing sedation and analgesia in the critically ill –A prospective cohort study	192 Patients	It's challenging to avoid over and under-sedation. Maintaining a target level of sedation is difficult; patients spend a large proportion of their ICU remaining at an insufficient level of sedation.
26	Patanwala et al., 2017,180 Days	Ketamine for analgosedation in the intensive care unit-A systematic review	6RCTs and 6 non-RCTs and 468 Patients	The use of ketamine may decrease the analgesic consumption in the intensive care unit. Additional studies are required to better define the role of ketamine for analgesia.
27	Patel SB et al., 2009,575 Days	Delirium and sedation in the intensive care unit (ICU)-A survey of behaviours and attitudes healthcare professionals	1384 Participants	Remifentanil requires less propofol but greater discomfort afterwards; equally successful sedation.Propofol faster wake-up, less days of MV, more efficient sedation.
28	Payen JF et al., 2007,391 Days	Current practices in sedation and analgesia for mechanically ventilated critically ill patients- A prospective multicenter Cohort	44 ICUs and 1381 Patients	Excessively deep sedation and lack of analgesia during painful operations must be avoided. Facilitate routine evaluation of pain and sedation and change the daily dose of drugs accordingly.
29	Pradilli L et al., 2017,1460 Days	Propofol or benzodiazepines for short-and long-term sedation in intensive care units- A Systematic review and met analysis	35 RCTs, 3015 Patients	Sedations are recommended with propofol than midazolam for short term sedation.
30	Reade MC et al., 2014, Follow up duration is not stated in the study	Sedation & delirium in the ICU-A prospective Cohort Study	418 Patients	Pain should be handled promptly and effectively, sedative administration should be kept to the minimum required for the comfort and protection of the patient, and early mobilization should be achieved wherever possible.
31	Rowe K et al., 2008, Follow up duration is not stated in the study	Continuing Education in Anesthesia, Critical Care & Pain	15 RCTs	Over-sedation can increase time on ventilator support, prolong ICU stay, and may Precipitate unnecessary neurological investigations.
32	Rozendaal et al., 2009, Follow up duration is not stated in the study	Remifentanil-propofol analgo-sedation shortens duration of ventilation and length of ICU stay compared to a conventional regimen- A randomized controlled trial	15 Hospitals and 205 Patients	In patients with predicted short-term duration of MV, remifentanil substantially improves sedation and agitation and decreases weaning time. This would lead to a shorter period of MV and ICU-LOS.
33	Schweickert et al., 2008,185 Days	Strategies to optimize analgesia and sedation – A randomized Controlled trails	132 Patients	Adequate but not excessive sedation in critically ill, mechanically ventilated patients is a complicated operation. The analgesics and sedatives used in this context are extremely potent, and drug and metabolism requirements are unpredictable.
34	Schweickert et al., 2009,30 Days	Early physical and occupational therapy in mechanically ventilated, critically ill patients- A randomized controlled trial	104 Patients	. Early detection and treatment of potential underlying causes of agitation and anxiety, such as pain, delirium, hypoxemia, hypoglycaemia, hypotension or alcohol withdrawal and other medications, are very critical prior to patient sedation.
35	Sessler et al., 2011, Follow up duration is not stated in the study	Protocolized and target-based sedation and analgesia in the ICU- A systematic review and met analysis	20 Randomized controlled trials and 3588 patients	Protocolized target-based sedation and analgesia are essential to successful sedation control. Significant components include the identification of targets and individual targets, the use of valid and reliable instruments to assess pain, agitation and sedation, and the titration of a logically selected combination of sedatives and analgesics to specified endpoints.
36	Sessler et al., 2008, Follow up duration is not stated in the study	Patient-focused sedation and analgesia in the ICU-A systematic review	53 Articles	Patient-focused treatment includes the selection of drugs ideally suited to patient characteristics, including the involvement of organ dysfunction that can affect drug metabolism or an unnecessary risk of side effects.
37	Shinotsuka et al., 2013, Follow up duration is not stated in the study	Perceptions and practices regarding delirium, sedation and analgesia in critically ill patients- A narrative review	39 Articles	Oversedation has been found to be dangerous and light sedation, and no-sedation procedures are correlated with enhanced patient outcomes.
38	Szumita et.al,2007, Follow up duration is not stated in the study	Sedation and analgesia in the intensive care unit evaluating the role of dexmedetomidin-A systematic review and met analysis	24 RCTs And 2160 patients	Dexmedetomidine can be an effective agent for sedation and analgesia in the ICU. However the lack of clinically significant endpoints in the trials, the concern about adverse cardiovascular effects and the relatively high acquisition cost of this medication reduce its use.
39	Tonner et al., 2003, Follow up duration is not stated in the study	Sedation and analgesia in the intensive care unit-A systematic review and met analysis	37 Articles and 4312 patients	Sedation and analgesia are now seen as an important part of intensive care treatment instead of being an inconvenient but required and minor problem.
40	Vincent et.al,2016, Follow up duration is not stated in the study	Comfort and patient-centred care without excessive sedation- A Systematic Review	74 Articles	Multimodal analgesia intended to reduce opioid use. Sedation is secondary to pain relief and where appropriate, should be dependent on agents that can be titrated to a defined target level that is subject to frequent examination and adjustment; the routine usage of benzodiazepines should be reduced.
41	Weinert et al., 2007,1095 Days	Epidemiology of sedation and sedation adequacy for mechanically ventilated patients in a medical and surgical intensive care unit- A prospective Cohort Study	274 Patients	While in 32% and 21% of sedation tests, patients were minimally arousable or non-arousable, interestingly, an oversedation rate of <3% occurred.
42	Woein et al., 2012,60 Days	Analgesia and sedation of mechanically ventilated patients– A national survey	54 ICUs and 108 participants	Potential factors that can enhance sedation and pain control of manually ventilated patients in Norwegian ICUs are more formal evaluation of pain and sedation and the use of written protocols. Strategies to minimize side effects should be approached
43	Yang HY et al., 2014,240 Days	Sufentanil for analgesia/sedation in patients in intensive care unit- A multicenter randomized controlled trial	11 Hospitals 544 Patients	The effectiveness of sufentanil analgesia is greater relative to fentanyl. Sufentanil has less physiological involvement and lower frequency of adverse reactions in patients with ICU.
44	Zalieckas et al., 2011, Follow up duration is not stated in the study	Sedation and analgesia in the ICU – A Systematic review and met analysis	39 Articles	Usage of sedation algorithms and emphasis on sedation protocols are necessary to reduce the total dosage and length of sedatives and analgesics used.

### 3.1 Areas of controversy regarding ICU sedation and analgesia

A variety of patients are now being admitted to the ICU for mechanical ventilation and other treatment approaches, but they are treated and intervened differently without any standard steps. It is clear that there is no evidence-based protocol or means of addressing these ICU patients that has stopped health practitioners from treating their patients stepwise and logically. Due to these and other conflicting factors patients in the ICU are treated differently irrespective of their disease and their need.

An RCT done on Perceptions and practices regarding delirium, sedation and analgesia in critically ill patients shows that over sedation has been shown to be dangerous and light sedation, and no sedation procedures are correlated with improved patient outcomes. In addition, deep sedation is frequently used to alleviate anxiety and facilitate amnesia in mechanically ventilated patients. In addition, deep sedation allows healthcare professionals to offer ICU patient treatment. However, unregulated administration of sedatives is sometimes correlated with over-sedation, which has been shown to increase the period of mechanical ventilations (18).

On the other hand, other literature strongly disagrees against light sedation and advises against implementing light sedation protocols as these results in accidental loss of endotracheal tubes and other instruments, increased anxiety, etc. Light sedation may enhance the pain and terrifying memories that survivors of ICU typically remember [19].

Deep sedation is also used to relieve anxiety and facilitate amnesia in mechanically ventilated patients. In addition, deep sedation allows healthcare professionals to offer ICU patient treatment [18,20].

Another big controversy and evidence-based practice here in our hospital is the use of benzodiazepines, but according to study guidelines, sedation methods using non-benzodiazepine should be favored over sedation with benzodiazepines to increase clinical results in ICU patients. However, the current literature reports modest differences in outcomes with benzodiazepine based versus non benzodiazepine-based sedation (3).

## 4 Discussions

A systematic review and meta-analysis of 13 RCTs showed that ICU sedation with volatile anesthetic agents relative to traditional intravenous sedatives, such as propofol or midazolam, shortened the duration of awakening and extubation. Despite these reductions in waking and extubation times with unpredictable sedation, no reductions in duration of stay in the ICU or hospital were noted. Compared to IV sedation, unpredictable sedation administered in the ICU shortened waking and extubation times (5).

Sedation protocols versus daily disruption of sedation, systematic study and meta-analysis.

Dedicates that sedation protocols and daily sedation interruption tend to be similar to techniques targeting lighter sedation levels, although it should be noted that the sedation target should be the primary objective of management in most patients under mechanical ventilation [21,22].

Other systematic reviews and meta-analysis indicate that there are no variations between sedation protocols targeting light sedation levels and daily sedation interruption strategies for mortality, duration of mechanical ventilation and duration of ICU stay. With the use of sedation procedures targeting lighter sedation levels, the number of days of free mechanical ventilation was higher and the hospital stay was shorter [23,24].

Randomized controlled trial done by Department of Critical Care, Peking University People's Hospital, Beijing, China clearly states that insufficient analgesia results in worsening stress, sleep deprivation, cognitive dysfunction, Anxiety and even delirium [25,26].Patients who received benzodiazepines have a relatively greater risk of delirium; analgesics can reduce the amount of sedatives required and can further reduce the occurrence of delirium And improve the prognosis [27].

Clonidine is a feasible alternative to midazolam without significant safety concerns. While both medications may cause withdrawal symptoms, patients who have been sedated with midazolam may need additional care for withdrawal after treatment [28].

A trial-based economic assessment shows that clonidine is likely to be a cost-effective sedative agent relative to midazolam. Neither drug in combination with traditional morphine will provide ideal sedation. Additional sedation, either with more than one medication or with another agent, is required to be sustained consistently at the targeted sedation stage. Maintaining individuals within tight confines of ideal sedation requires frequent evaluation and the ability to provide rapid rescue sedation [29].

### 4.1 Regarding sedation scales in the ICU

The majority of mechanically ventilated patients need sedation. Preventing unnecessary deep sedation is a priority in Intensive Care Units (ICUs), associated with adverse effects such as longer ICU stays, more ICU infections and higher mortality. Lighter sedation can improve these results, but anxiety can also endanger protection and increase the workload and tension of workers. Lighter sedation often theoretically exposes patients to pain and distress reported by ICU survivors [20,30].

Optimum sedation is unique to the patient, but the prevention of deep sedation should be considered when maintaining appropriate control of pain and agitation. The most successful system-level methods for maximizing all aspects of sedation within ICUs are unclear and introducing and maintaining changes in ICU sedation quality are difficult [31,32].

A standardized tool for evaluating sedation and agitation is required to track sedation levels. It helps to titrate sedatives and to determine agitated behavior, even though all sedation scales have their own limits, the 2013 clinical guideline for pain, agitation and delirium in adult ICU patients has shown that the Richmond agitation sedation scale (RASS) and (SAS) are the most accurate and effective sedation evaluation scales for sedation depth and consistency measurement (16).

In a study comparing the validity and reliability of RASS with the SAS scale, the final result showed that RASS is reasonable, easy to remember and simple to administer, and that RASS also has high validity and reliability in surgical and medical patients, in ventilated and non-ventilated patients for sedated and non-sedated adult ICU patients. It also defined that RASS had advantages in reducing the dose of sedative medication and the duration of mechanical ventilation (17).

Evidence is increasing that volatile anesthetic agents are associated with faster extubation times, better cardiovascular stability with no end-organ toxicity relative to our normal intravenous agents for short-term critical care sedation. The use of volatile agents in the ICU is a novel strategy that uses a specialized distribution and scavenging method that needs personnel training and cultural acceptance. Compared to IV sedation, ICU short-term volatile sedation is administered by ACD in the ICU shortened awakening and extubation times [33,34]. Considering the difference in serum troponin levels between both arms, volatile anesthetics might have myocardial protective effect after cardiac surgery even at a sub anesthetic dose (5).

Sedatives are given to 85% of patients in the Intensive Care Unit (ICU). The sedatives most widely used are intravenous benzodiazepines and propofol. These agents are associated with over-sedation in 40–60% of patients, which may lead to prolonged intubation, delirium and drug-induced hypotension (18).

### 4.2 Regarding pain assessment and analgesia in the ICU

Pain is one of the causes of anxiety for critically ill patients and can be a positive indication or warning for some of the pathophysiological issues that need to be corrected, but also a bad cause of unnecessary stressors for certain pathophysiological problems, but it is difficult to quantify pain in the ICU, particularly for those who are not aware or unable to talk [35]. So several tools for measuring the pain of critically ill patients has been already developed and validated. A prospective observational study showed that for uncommunicating patients the commonly and best pain assessment tools used are BPS (Behavior Pain Scale) and CPOT (Critically-ill Pain Observation Tool) [3].

BPS use three parameters that are facial expression, upper extremity movement, and the compliance with ventilator, while CPOT use four parameters that are facial expression, muscular tone (passive movement), upper extremity movement (active), and the compliance with the ventilator. Study indicates that CPOT and BPS showed a strong criterion and distinguish validity (p < 0.0001). BPS was found to be more specific (91.7%) than CPOT (70.8%), but less sensitive (BPS 62.7%, CPOT 76.5%). COPT and BPS scores were significantly correlated with VAS (p < 0.0001). The combination of BPS and CPOT resulted in better sensitivity 80.4% For conscious patients who can self-report VAS is the gold standard for evaluation of pain and VAS≥3 is used to determine patient with pain [11].

Choice of powerful sedatives and analgesic medications is clearly of importance to our patients’ clinical outcomes [36]. In addition to deciding how to dose and titrate, and when we chose to discontinue these drugs, it is of utmost importance. Increased attention has recently been paid to adequate titration of sedative and analgesic drugs in critically ill patients, in particular those treated with mechanical ventilation [37]. Patient comfort should be a primary goal in the intensive care unit (ICU), including adequate pain control, anxiolysis, and prevention and treatment of delirium. However, adequate balance of sedation and analgesia is difficult. Without rational and accepted target levels of sedation, it is possible that various health team members may have disparate treatment priorities, increase the risk of iatrogenic complications and possibly delay recovery (3).

With regard to sedation in the ICU, it is important to note that the treatment of mechanically ventilated patients under the heading of sedation must first understand the need for adequate regulation of pain. Pain is a condition mostly encountered by critically ill patients [38]. Pain can be experienced as a consequence of intubation and mechanical ventilation itself, or it can be a consequence of other routine clinical care such as moving a patient in bed or adjusting tubes and lines. Pain can be substantial and initiate elements of the stress response. Pain should also be treated in order to ensure patient satisfaction and potentially reduce accompanying adverse events. It is possible that patients with adequate pain control may require few or no sedatives, as noted in the Danish study, although the importance of attention to pain is undeniable, it is equally important to recognize that not all mechanically ventilated patients in the ICU are actually experiencing pain [6,11,39].

Patients at high risk of dying and pain are interviewed for up to 2 weeks after their ICU experience. This research is significant because it indicates that while universal consideration of the likelihood of pain is required, there is no need for a universal analgesic administration strategy [[40], [41], [42]]. The best way to approach analgesia in mechanically ventilated patients in the ICU is to interact directly with the patient [2].

## 5 Conclusion

Analgesia and sedation are important therapies in the ICU [11,30]. ICU patients have a delirium rate of up to 80%, in addition to increased mortality, longer hospital stays, higher hospital costs and poor long-term outcomes (3).

Sedation protocols and daily sedation interruption do not appear to differ in regard to the majority of analyzed outcomes [43,44]. The only differences observed were small and had a high degree of heterogeneity [17,20]. The key indications for the use of these sedatives and analgesics include: To reduce patient discomfort, to avoid anxiety and agitation, to cause amnesia, to promote mechanical ventilation, to prevent accidental displacement of endotracheal tubes, and to reduce cell metabolism and so on [15].

Pain should be evaluated and handled appropriately for patients in ICU(40). The final goal of sedation is to have an awake and alert patient who could perform weaning trials according to each respected ICU protocols [19].

### 5.1 Recommendations

For pain assessment and analgesia, it is recommended that a combination of BPS be used; it is recommended that light sedation with daily interruption of sedative infusion or titration of sedative dose be required for the final purpose of awakening and alertness of a patient who can conduct a weaning test if there is no contraindication. It is also strongly advised that the use of RASS is an important sedation assessment tool in adult ICU patients and the implementation of a revised ICU analgesia, sedation and delirium protocol has been associated with enhanced RASS and RSS assessment and documentation; reduced hourly benzodiazepine dose; and decreased delirium and median durations of mechanical ventilation, ICU stay, and hospitalization [9].

As regards the pharmacological option of sedatives, Propofol is strongly recommended for short-term sedation and is superior to midazolam and other sedatives. It is just as effective for medium and long-term sedation as midazolam for more than 72 h. So we can use Propofol for both short-term and long-term sedation methods safely (45). It is again recommended that the use of ketamine as an alternative sedative agent in adult ICU patients is very necessary, particularly in patients with asthma and hypotensive blood pressure and CPOT in critically ill, mechanically ventilated adult ICU patients. And for a conscious adult ICU patient, it is advised to use VAS for validated pain assessment [18,20].

### 5.2 Limitations and challenge

This review article had its limitation and challenges. The authors thought that the limitations are acceptable and challenges are over come accordingly. Luck of very recent studies and using of different article types was one limitation and challenges which has been overcome by taking studies done in the past 20 years and considering recommendation.

Availability of a variety of sedation and pain assessment tools and differences in utilization of these tools set up to set up was another limitation and challenge and we have overcome it by just taking the most widely used tools and by localizing the guideline to a resource limited localized set up.

### 5.3 Summary of sedation and pain assessment tools

In this systematic review and evidence based guideline different sedation assessment tools have been used, of which the Blooms Burry Sedation scale is the one. In the Blooms Burry Sedation patients having sedation scores −3 up to 2 do not need any sedative, while those who have 3 and above are in need of sedatives accordingly (Table 3).

**Table 3:** Blooms Burry sedation scale (BBSS).

Seesdation Scors	Behavior of the patient
3	Agitated and restless
2	Awake and comfortable
1	Aware but calm
0	Roused by voice
−1	Roused by touch
−2	Roused by painful stimuli
−3	Unrousable
A	A Natural sleep
P	Paralysed

For critically ill patients we can use behavioral pain Scales. The tool principally considers Facial expression, Limbic movement and Complains with a mechanical ventilators (Table 4).

**Table 4:** Behavioral pain scale (BPS).

Items	Description	Scores
FACIAL EXPRESSION	Relaxed	1
	Partially tightened … (Brow lowering)	2
	Fully tightened …… (Eyelid closing)	3
	Grimacing	4
UPPER LIMBS	No movement	1
	Partially bent	2
	Fully bent with finger flexion	3
	Permanently retracted	4
COMPLIANS WITH VENTILATION	Tolerating Movement	1
	Coughing but tolerating ventilation most of the time	2
	Fighting ventilator	3
	Unable to control ventilation	4

The sedation-Agitation Scale a very important tool to assess both levels of sedation and agitation. In this tool scores 1–2 are for unawake patients and doesn't request use of sedative, while 3–7 are awake patients of which 5–7 are in need of Sedative use (Table 5).

**Table 5:** Sedation-agitation scale (SAS).

Score	Description	State
7 6 5 4 3	Dangerous agitation	A W A K E
	Very agitated	
	Agitated	
	Calm and Cooperative	
	Sedated	
2	Very Sedated	N O T A W A K E
1	Unarousable	

The Richmond Agitation Sedation Scale the most important tool that is frequently used. Patients who have score of 1–4 are in state of restless to combative and needs sedation. Those having scores -5 – 0 are in state of unarousable to alert and calm so that do not need sedation (Table 6).

**Table 6:** Richmond agitation sedation scale (RASS).

Score	Terms	Description
+4	Combative	Overtly combative or violent; immediate danger to staff
+3	Very agitated	Pulls on or removes tube(s) or catheter(s) or has aggressive behavior towards staff
+2	Agitated	Frequent nonpurposeful movement or patient–ventilator dyssynchrony
+1	Restless	Anxious or apprehensive but movements not aggressive or vigorous
0	Alert& calm	Considered Normal and obeys commands
−1	Drowsy	Not fully alert, but has sustained (more than 10 s) awakening, with eye contact in response to voice
−2	Light sedation	Briefly (less than 10 s) awakens with eye contact in response to voice
−3	Moderate sedation	Any movement (but no eye contact) in response to voice
−4	Deep sedation	No response to voice, but any movement in response to physical stimulation
−5	Unarousable	No response to voice or physical stimulation

For the assessment of pain in critically ill patients we preferentially use the Critically Ill Pain Observation Tool. This tool considers facial expression, body movement, muscle tension, copmlaince with mechanical ventilators and vocalization for extubated patients (Table 7).

**Table 7:** Critically ill pain observation tool (CPOT).

Indicators	Descriptions	Scores
FACIAL EXPRESSION	RELAXED, NUETRAL	0
	TENSE	1
	GRIMACING	2
BODY MOVEMENT	ABSENCE OF MOVEMENTs	0
	PROTECTION	1
	RESTLESSNESS	2
MUSCLE TENSION	RELAEDX	0
	TENSE OR RIGID	1
	VERY TENSE/RIGID	2
COMLIANS WITH VENITLATOR (INTUBAED PTs)	TOLARATING VENTILATOR	0
	COUGHING, BUT TOLERATEs	1
	FIGHTING VENTILATOR	2
VOCALIZATION (EXTUBATED PTs)	TALKING IN NORMAL TONE	0
	SIGHING, MAONING	1
	CRYING OUT,SOBBING	2

**Fig. 2:**
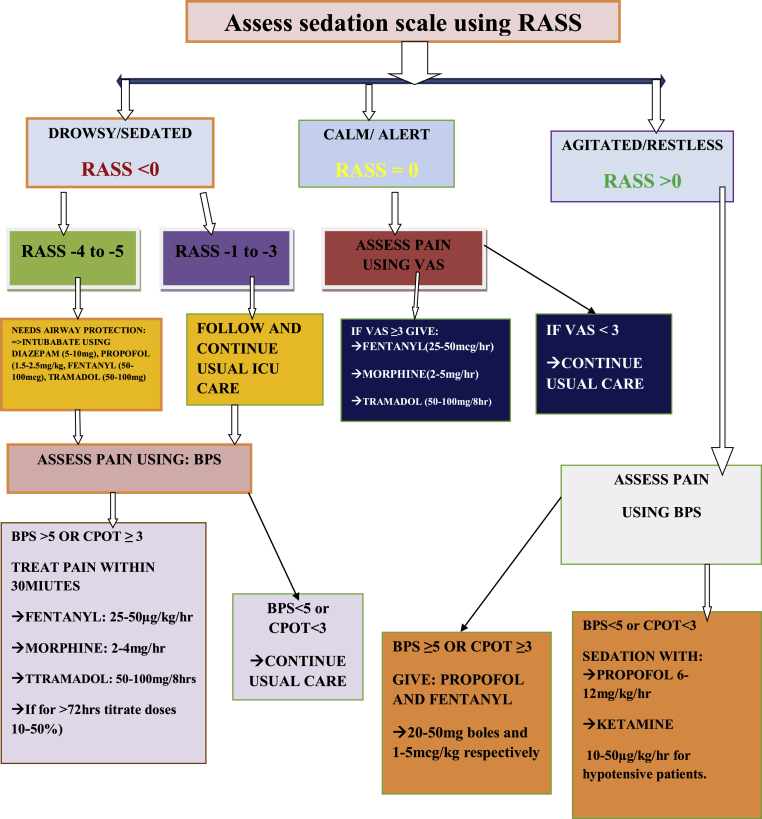
Practice guideline on adult sedation and analgesia for a resource limited ICU settings.

Finally flow diagram was drawn based the collected information's from the literatures. The flaw is made after it has been contextualized into limited setups (Fig. 2).

## Declaration

The authors declares that this is an original work of the authors.

## Ethical approval

Not required.

## Consent for publication

Not applicable for this publication.

## Competing of interest

All the authors declared that there is no competing of interest.

## Funding

None.

## Please state any conflicts of interest

The authors declared that there is no conflict of interest.

## Please state any sources of funding for your research

No funding is required

## Consent

Not applicable for that.

## Registration of Research Studies

1. Name of the registry: http://www.researchregistry.com

2. Unique Identifying number or registration ID: researchregistry 6620

3. Hyperlink to your specific registration (must be publicly accessible and will be checked: https://www.researchregistry.com/browse-the registry#home/registrationdetails /603cbc8873c40d001b3a44f1/.

## Guarantor

Mr. Netsanet Temesgen

## Authors' contributions

NT, BC, TT, DE, NS and AB performed literature search, assessment of articles, data extraction, data analysis, and manuscript preparation and all the authors have read and approved the manuscript well.

## Abbreviations and Acronym

ASA: American society of anaesthesiologists
BPS: Behavioral pain scale
CPOT: Critical care pain observation tool
GURH: Gondar university referral hospital
ICU: Intensive care unit
RASS: Richmond agitation sedation scale
RCT: Randomized controlled trail
RSS: Ramsay sedation scale
SAS: Sedation agitation scale
VAS: Visual analogue score

## References

[1] Zalieckas J., Weldon C. (2015). Sedation and Analgesia in the ICU. Seminars in Pediatric Surgery.

[2] Keogh S.J., Long D.A., Horn D.V. (2015). Practice guidelines for sedation and analgesia management of critically ill children: a pilot study evaluating guideline impact and feasibility in the PICU. BMJ Open.

[3] Kress J.P., Pohlman A.S., Hall J.B. (2002). Sedation and analgesia in the intensive care unit. Am. J. Respir. Crit. Care Med..

[4] Lavrentieva A., Depetris N., Rodini I. (2017). Analgesia, sedation and arousal status in burn patients: the gap between recommendations and current practices. Annals of Burns and Fire Disasters.

[5] Fraser G.L., Devlin J.W., Worby C.P., Alhazzani W., Barr J., Dasta J.F. (2013). Benzodiazepine versus nonbenzodiazepine-based sedation for mechanically ventilated, critically ill adults: a systematic review and meta-analysis of randomized trials. Read. Online: Critical Care Medicine| Society of Critical Care Medicine.

[6] Tonner P.H., Weiler N., Paris A., Scholz J. (2003). Sedation and analgesia in the intensive care unit. Curr. Opin. Anesthesiol..

[7] Kim H.Y., Lee J.E., Kim H.Y., Kim J. (2017). Volatile sedation in the intensive care unit: a systematic review and meta-analysis. Medicine.

[8] Weinert C.R., Calvin A.D. (2007). Epidemiology of sedation and sedation adequacy for mechanically ventilated patients in a medical and surgical intensive care unit. Crit. Care Med..

[9] Wøien H., Stubhaug A., Bjørk I. (2012). Analgesia and sedation of mechanically ventilated patients–a national survey of clinical practice. Acta Anaesthesiol. Scand..

[10] Fraser G.L., Riker R.R. (2007). Sedation and analgesia in the critically ill adult. Curr. Opin. Anesthesiol..

[11] Vincent J.-L., Shehabi Y., Walsh T.S., Pandharipande P.P., Ball J.A., Spronk P. (2016). Comfort and patient-centred care without excessive sedation: the eCASH concept. Intensive Care Med..

[12] Ely E.W., Truman B., Shintani A., Thomason J.W., Wheeler A.P., Gordon S. (2003). Monitoring sedation status over time in ICU patients: reliability and validity of the Richmond Agitation-Sedation Scale (RASS). Jama.

[13] Mukhopadhyay A., Tai B.C., Remani D., Phua J., Cove M.E., Kowitlawakul Y. (2017). Age related inverse dose relation of sedatives and analgesics in the intensive care unit. PloS One.

[14] Devabhakthuni S., Armahizer M.J., Dasta J.F., Kane-Gill S.L. (2012). Analgosedation: a paradigm shift in intensive care unit sedation practice. Ann. Pharmacother..

[15] Devlin J.W., Roberts R.J. (2009). Pharmacology of commonly used analgesics and sedatives in the ICU: benzodiazepines, propofol, and opioids. Crit. Care Clin..

[16] Reade M.C., Finfer S. (2014). Sedation and delirium in the intensive care unit. N. Engl. J. Med..

[17] Rowe K., Fletcher S. (2008). Sedation in the intensive care unit.

[18] Shinotsuka C.R., Salluh J.I.F. (2013). Perceptions and practices regarding delirium, sedation and analgesia in critically ill patients: a narrative review. Revista Brasileira de Terapia Intensiva.

[19] Baron R., Binder A., Biniek R., Braune S., Buerkle H., Dall P. (2015). Evidence and consensus based guideline for the management of delirium, analgesia, and sedation in intensive care medicine. Revision 2015 (DAS-Guideline 2015)–short version. Ger. Med. Sci..

[20] Schweickert W.D., Kress J.P. (2008). Strategies to optimize analgesia and sedation. Crit. Care.

[21] MacLaren R., Plamondon J.M., Ramsay K.B., Rocker G.M., Patrick W.D., Hall R.I. (2000). A prospective evaluation of empiric versus protocol‐based sedation and analgesia. Pharmacotherapy.

[22] Martin J., Franck M., Fischer M., Spies C. (2006). Sedation and analgesia in German intensive care units: how is it done in reality? Results of a patient-based survey of analgesia and sedation. Intensive Care Med..

[23] Brush D.R., Kress J.P. (2009). Sedation and analgesia for the mechanically ventilated patient. Clin. Chest Med..

[24] Burry L., Rose L., McCullagh I.J., Fergusson D.A., Ferguson N.D., Mehta S. (2014). Daily sedation interruption versus no daily sedation interruption for critically ill adult patients requiring invasive mechanical ventilation. Cochrane Database Syst. Rev..

[25] Martin J., Parsch A., Franck M., Wernecke K.D., Fischer M., Spies C. (2005). Practice of sedation and analgesia in German intensive care units: results of a national survey. Crit. Care.

[26] Meiser A., Laubenthal H. (2005). Inhalational anaesthetics in the ICU: theory and practice of inhalational sedation in the ICU, economics, risk-benefit. Best Pract. Res. Clin. Anaesthesiol..

[27] Hutton B., Burry L.D., Kanji S., Mehta S., Guenette M., Martin C.M. (2016). Comparison of sedation strategies for critically ill patients: a protocol for a systematic review incorporating network meta-analyses. Syst. Rev..

[28] Park G., Coursin D., Ely E.W., England M., Fraser G.L., Mantz J. (2001). Commentary. Balancing sedation and analgesia in the critically ill. Crit. Care Clin..

[29] Yang H., Sun R., Chang Y., Fu Y., Li B., Qin B. (2014). A multicenter randomized controlled trial of sufentanil for analgesia/sedation in patients in intensive care unit. Zhonghua wei zhong bing ji jiu yi xue.

[30] Owen G.D., Stollings J.L., Rakhit S., Wang L., Yu C., Hosay M.A. (2019). International analgesia, sedation, and delirium practices: a prospective cohort study. J Intensive Care.

[31] Barr J., Fraser G.L., Puntillo K., Ely E.W., Gélinas C., Dasta J.F. (2013). Clinical practice guidelines for the management of pain, agitation, and delirium in adult patients in the intensive care unit. Crit. Care Med..

[32] Jerath A., Ferguson N.D., Steel A., Wijeysundera D., Macdonald J., Wasowicz M. (2015). The use of volatile anesthetic agents for long-term critical care sedation (VALTS): study protocol for a pilot randomized controlled trial. Trials.

[33] Patanwala A.E., Martin J.R., Erstad B.L. (2017). Ketamine for analgosedation in the intensive care unit: a systematic review. J. Intensive Care Med..

[34] Pradelli L., Povero M., Bürkle H., Kampmeier T.-G., Della-Rocca G., Feuersenger A. (2017). Propofol or benzodiazepines for short-and long-term sedation in intensive care units? An economic evaluation based on meta-analytic results. Clin. Outcomes Res.: CEOR.

[35] Dale C.R., Kannas D.A., Fan V.S., Daniel S.L., Deem S., Yanez N.D. (2014). Improved analgesia, sedation, and delirium protocol associated with decreased duration of delirium and mechanical ventilation. Ann Am Thorac Soc.

[36] Szumita P.M., Baroletti S.A., Anger K.E., Wechsler M.E. (2007). Sedation and analgesia in the intensive care unit: evaluating the role of dexmedetomidine. Am. J. Health Syst. Pharm..

[37] Ahlers S.J., van Gulik L., van der Veen A.M., van Dongen H.P., Bruins P., Belitser S.V. (2008). Comparison of different pain scoring systems in critically ill patients in a general ICU. Crit. Care.

[38] Deffland M., Spies C., Weiss B., Keller N., Jenny M., Kruppa J. (2020). Effects of pain, sedation and delirium monitoring on clinical and economic outcome: a retrospective study. PloS One.

[39] Schweickert W.D., Pohlman M.C., Pohlman A.S., Nigos C., Pawlik A.J., Esbrook C.L. (2009). Early physical and occupational therapy in mechanically ventilated, critically ill patients: a randomised controlled trial. Lancet.

[40] Sessler C.N., Pedram S. (2011). Protocolized and target-based sedation and analgesia in the ICU. Anesthesiol. Clin..

[41] Sessler C.N., Varney K. (2008). Patient-focused sedation and analgesia in the ICU. Chest.

[42] Patel R.P., Gambrell M., Speroff T., Scott T.A., Pun B.T., Okahashi J. (2009). Delirium and sedation in the intensive care unit (ICU): survey of behaviors and attitudes of 1,384 healthcare professionals. Crit. Care Med..

[43] Payen J.-F., Chanques G., Mantz J., Hercule C., Auriant I., Leguillou J.-L. (2007). Current practices in sedation and analgesia for mechanically ventilated critically ill patients: a prospective multicenter patient-based study. J. Am. Soc. Anesthesiol..

[44] Rozendaal F.W., Spronk P.E., Snellen F.F., Schoen A., van Zanten A.R., Foudraine N.A. (2009). Remifentanil-propofol analgo-sedation shortens duration of ventilation and length of ICU stay compared to a conventional regimen: a centre randomised, cross-over, open-label study in The Netherlands. Intensive Care Med..

